# Supplementary material for: Tau is an m6A-associated RNA binding protein that causes RNA dysregulation in disease
Source: bioRxiv. 2026 Oct 29:2026.10.26.762095. Preprint. [Version 1] doi: 10.64898/2026.10.26.762095 (PMC13733919; doi:10.64898/2026.10.26.762095)
Supplement: Supplementary file 1 [file NIHPP2026.10.26.762095V1-supplement-1.pdf]

bioRxiv preprint doi: https://doi.org/10.64898/2026.10.26.762095; this version posted October 29, 2026. The copyright holder for this preprint (which was not certified by peer review) is the author/funder, who has granted bioRxiv a license to display the preprint in perpetuity. It is made available under a CC-BY 4.0 International license.

Figure S1

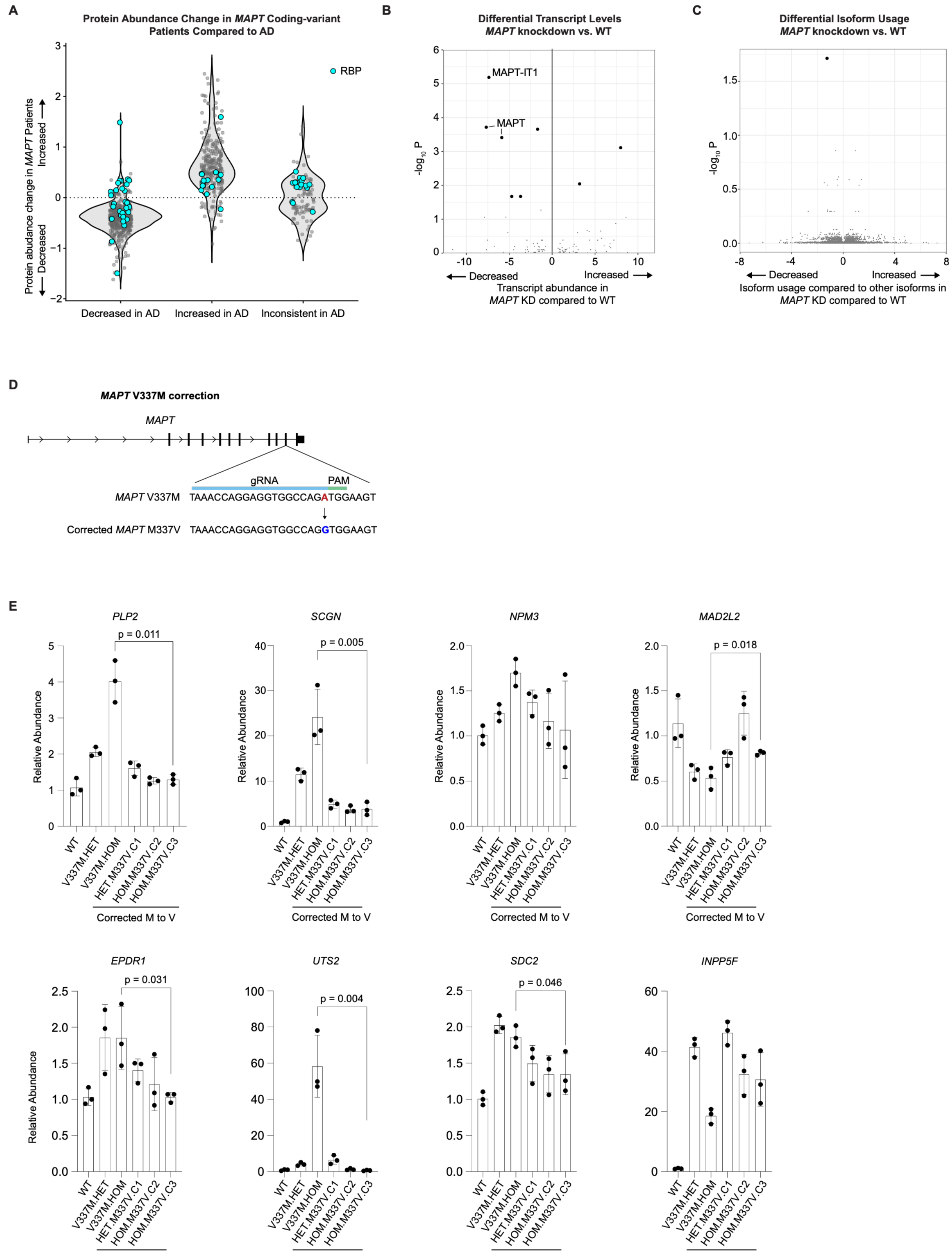

1192

bioRxiv preprint doi: https://doi.org/10.64898/2026.10.26.762095; this version posted October 29, 2026. The copyright holder for this preprint (which was not certified by peer review) is the author/funder, who has granted bioRxiv a license to display the preprint in perpetuity. It is made available under a CC-BY 4.0 International license.

Figure S1: FTD-associated *MAPT* mutations cause dysregulation of RNA-related processes. (A) Violin plot showing protein abundance changes in postmortem brain samples from *MAPT* coding-variant patients for proteins that are consistently decreased (left) and increased (right) in AD[26]. y-axis = $\log_2$(*MAPT* patient protein abundance / healthy control protein abundance). RBPs are highlighted. **(B)** Scatter plot of differentially abundant transcripts from long-read RNA sequencing for *MAPT* knockdown iNeurons compared to WT control. x-axis = $\log_2$(*MAPT* knockdown transcript abundance / *MAPT* WT transcript abundance). **(C)** Scatter plot of differentially used isoforms from long-read RNA sequencing for *MAPT* knockdown iNeurons compared to WT control. x-axis = (log-odds of transcript usage) − (log-odds of the other transcripts usage of the same gene). **(D)** Schematic for gene-editing of *MAPT* V337M iPSCs to *MAPT* M337V corrected lines. **(E)** qPCR for DEGs in *MAPT* V337M iNeurons for WT, heterozygous *MAPT* V337M (V337M.HET), homozygous *MAPT* V337M (V337M.HOM), *MAPT* M337V corrected from heterozygous *MAPT* V337M (HET.M337V.C), and *MAPT* M337V corrected from homozygous *MAPT* V337M (HOM.M337V.C) iNeurons. p-values were determined using student t-test. n = 3 replicates from separate wells of a differentiation. Error bars = standard deviation from the mean.

bioRxiv preprint doi: https://doi.org/10.64898/2026.10.26.762095; this version posted October 29, 2026. The copyright holder for this preprint (which was not certified by peer review) is the author/funder, who has granted bioRxiv a license to display the preprint in perpetuity. It is made available under a CC-BY 4.0 International license.

Figure S2

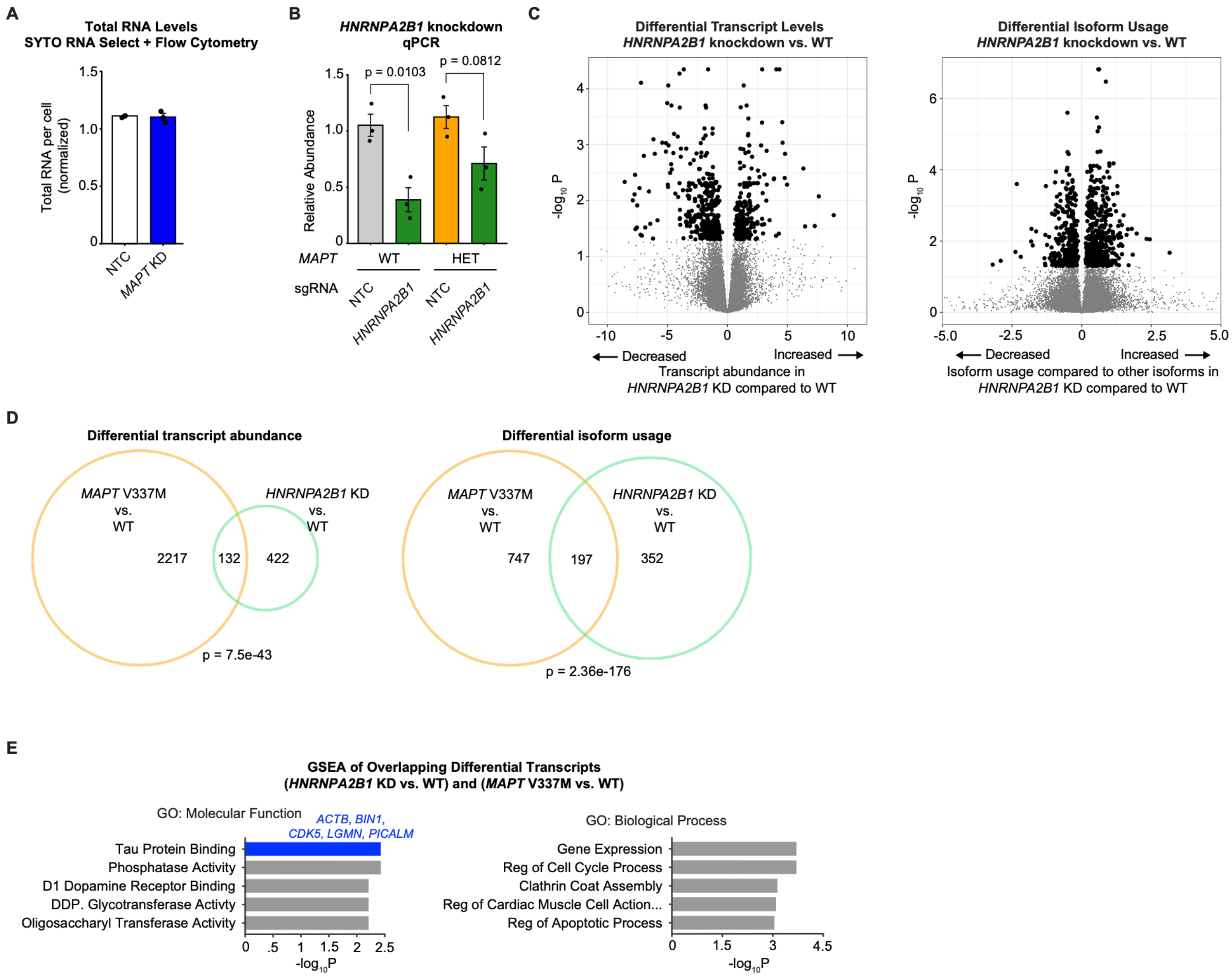

bioRxiv preprint doi: https://doi.org/10.64898/2026.10.26.762095; this version posted October 29, 2026. The copyright holder for this preprint (which was not certified by peer review) is the author/funder, who has granted bioRxiv a license to display the preprint in perpetuity. It is made available under a CC-BY 4.0 International license.

Figure S2: CRISPRi screen identifies hnRNPA2B1 as a modifier of total RNA levels in *MAPT* V337M iNeurons. (A) Total cellular RNA levels (measured by flow cytometry using SYTO RNA Select stain) for *MAPT* knockdown and WT control iNeurons. y-axis = (MFI of denoted genotype iNeurons / MFI of in-well control WT iNeurons). n = 3 replicates from separate wells of a differentiation. **(B)** qPCR for *HNRNPA2B1* in *MAPT* WT and *MAPT* V337M *HNRNPA2B1* knockdown iNeurons. p-values were determined using student t-test. n = 3 replicates from separate wells of a differentiation. Error bars = standard deviation from the mean. **(C)** Volcano plots of differentially abundant transcripts (left) and differentially used isoforms (right) from long-read RNA sequencing for *HNRNPA2B1* knockdown iNeurons compared to WT control. **(D)** Venn diagrams showing overlap between differentially abundant transcripts (top) and differentially used isoforms (bottom) for *MAPT* V337M and *HNRNPA2B1* knockdown iNeurons. p-values were determined through Fisher's exact test. (**E**) GSEA of overlapping differential transcripts from *MAPT* V337M and *HNRNPA2B1* knockdown iNeurons.

bioRxiv preprint doi: https://doi.org/10.64898/2026.10.26.762095; this version posted October 29, 2026. The copyright holder for this preprint (which was not certified by peer review) is the author/funder, who has granted bioRxiv a license to display the preprint in perpetuity. It is made available under a CC-BY 4.0 International license.

Figure S3

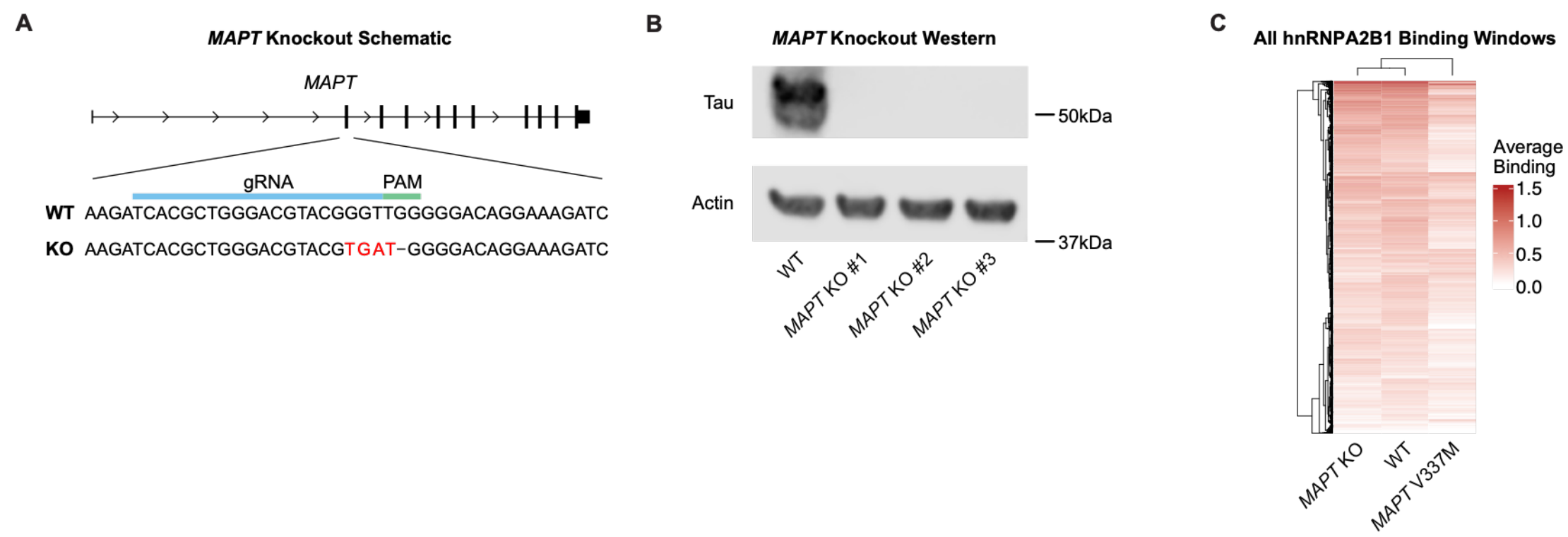

Figure S3: hnRNPA2B1 mRNA binding is dysregulated in *MAPT* V337M iNeurons. **(A)** Schematic of MAPT knockout, using CRISPR Cas9 gene editing. **(B)** Western blot validation of *MAPT* knockout using tau13 antibody. Three independent knockout clones are shown. **(C)** Heatmap of average hnRNPA2B1 binding at all binding windows detected in *MAPT* WT iNeurons from eCLIP experiment outlined in **Figure 3A**.

bioRxiv preprint doi: https://doi.org/10.64898/2026.10.26.762095; this version posted October 29, 2026. The copyright holder for this preprint (which was not certified by peer review) is the author/funder, who has granted bioRxiv a license to display the preprint in perpetuity. It is made available under a CC-BY 4.0 International license.

Figure S4

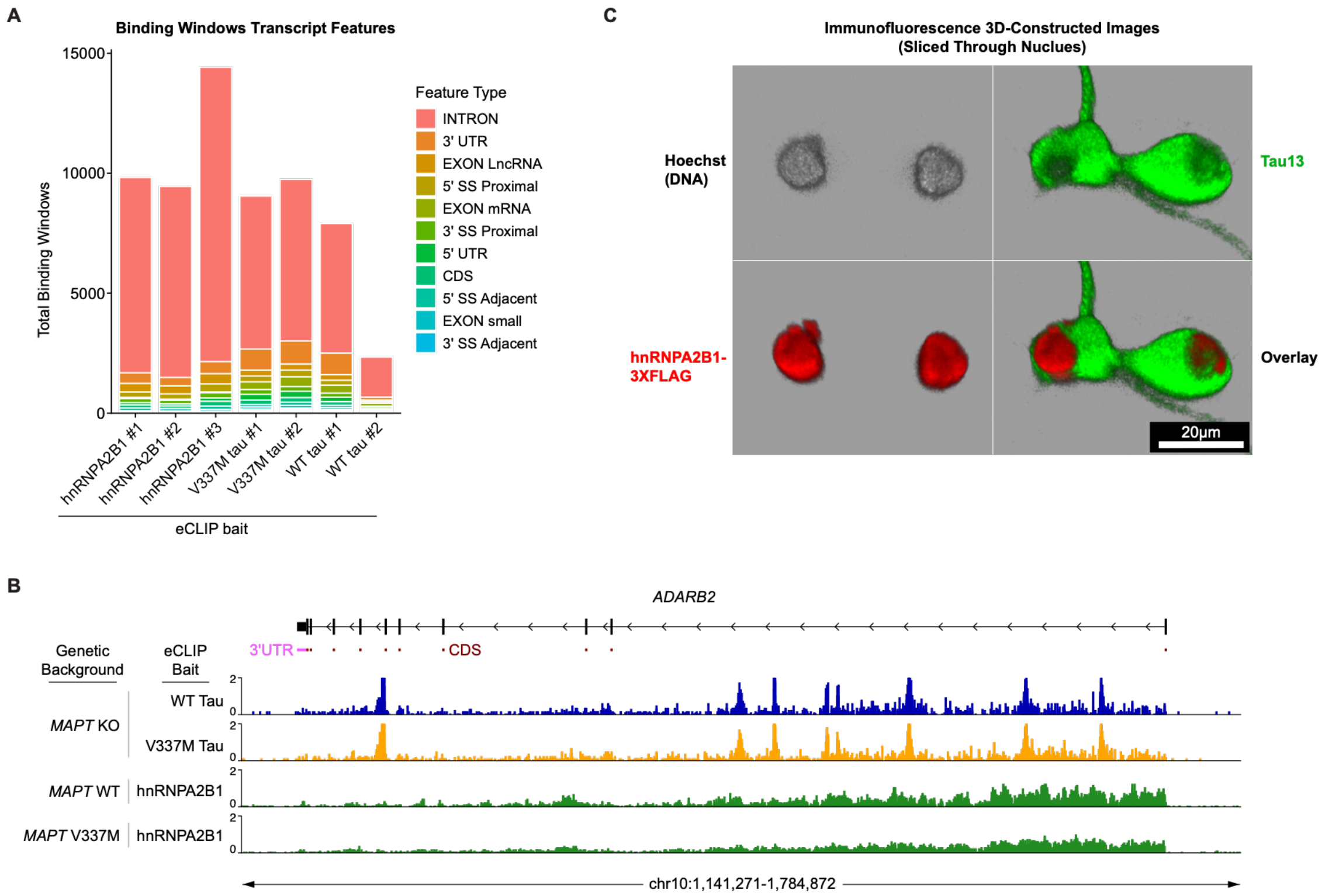

Figure S4: Tau binds to RNA with a similar profile to hnRNPA2B1. (A) Stacked bar plot showing the distribution of transcript features at binding windows for denoted factors for separate eCLIP replicates. **(B)** Genomic tracks showing eCLIP binding intensity (log2-fold change of IP signal over input) for WT-tau and hnRNPA2B1 in *MAPT* WT iNeurons. **(C)** 3D immunofluorescence images cross sectioned through the center of nuclei, imaging for GFP-tau and hnRNPA2B1-3XFLAG in WT iNeurons. Nuclear DNA is stained with Hoechst.

bioRxiv preprint doi: https://doi.org/10.64898/2026.10.26.762095; this version posted October 29, 2026. The copyright holder for this preprint (which was not certified by peer review) is the author/funder, who has granted bioRxiv a license to display the preprint in perpetuity. It is made available under a CC-BY 4.0 International license.

Figure S5

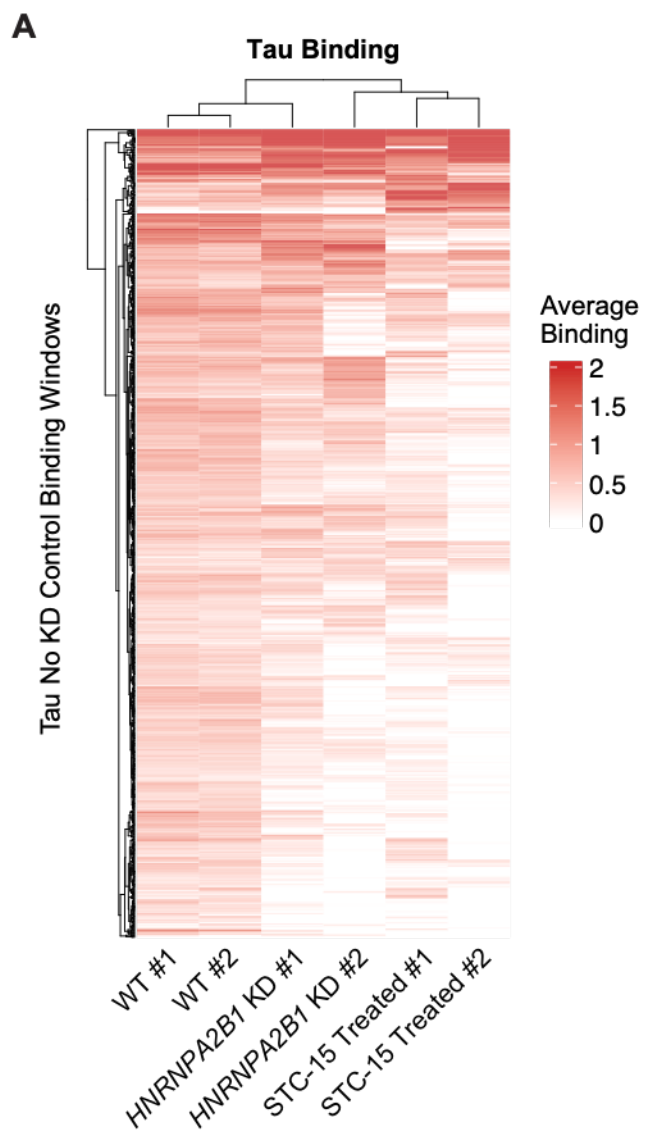

Figure S5: hnRNPA2B1 and m^{6}A promote the binding of tau with RNA.

(A) Heatmap showing average binding levels at WT control tau-binding windows for all eCLIP replicates (experiment outlined in Fig. 5D) for No-KD targeting control, *HNRNPA2B1* KD, and STC-15 treated iNeurons.

bioRxiv preprint doi: https://doi.org/10.64898/2026.10.26.762095; this version posted October 29, 2026. The copyright holder for this preprint (which was not certified by peer review) is the author/funder, who has granted bioRxiv a license to display the preprint in perpetuity. It is made available under a CC-BY 4.0 International license.

Figure S6

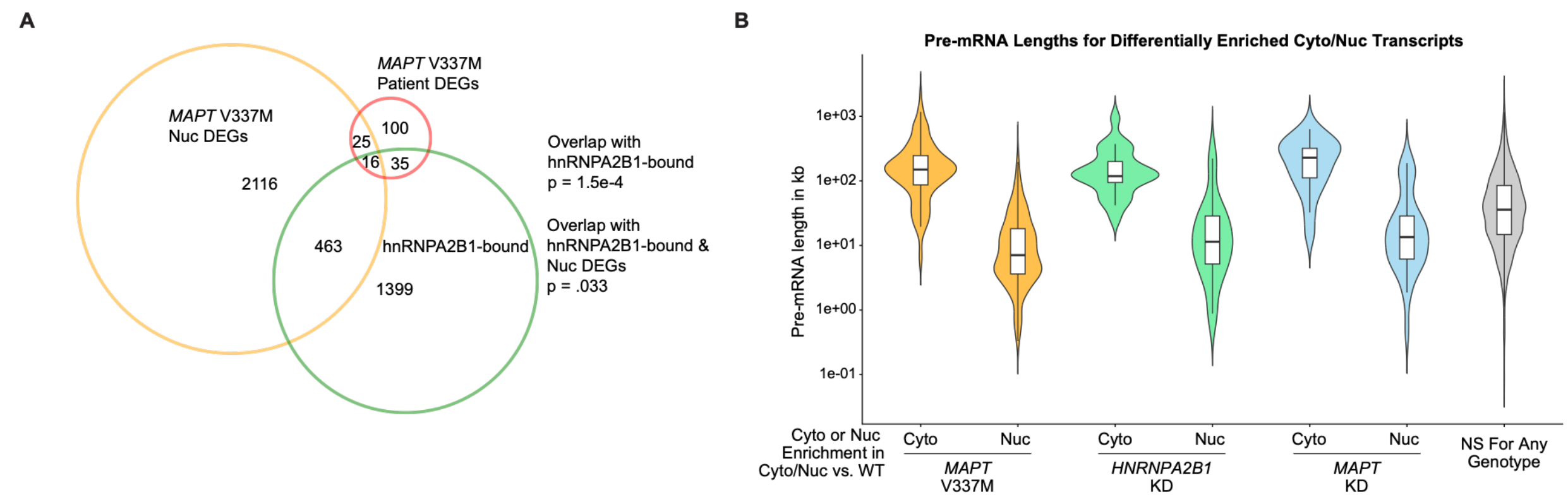

Figure S6: Patterns of cytosolic and nuclear mRNA changes suggest compartment-specific regulation. (A) Venn diagrams for overlap between differential genes from *MAPT* V337M postmortem brain sample transcript profiling[41], hnRNPA2B1-bound transcripts, and nuclear DEGs from *MAPT* V337M compared to WT iNeurons. p-values represent enrichment for *MAPT* V337M patient DEGs relative to the non-significant transcript background, using Fisher's exact test. **(B)** Violin plots showing maximal pre-mRNA lengths for transcripts with differential cyto/nuc abundance ratio compared to WT.
